# Incidence and risk factors associated with negative postoperative behavioral changes in children undergoing painless gastroscopy

**DOI:** 10.1186/s12887-023-04187-8

**Published:** 2023-07-20

**Authors:** Pu YanYing, Liu ShenLing, Peng XiaoHan, Xu YunBo, Tan Xin, Li GuoYan, Cheng Yan, Huang Lei

**Affiliations:** Sedation and Analgesia Center, Kunming Children’s Hospital, Kunming, China

**Keywords:** Pediatric, Anesthesia, Child behavior, Postoperative, Negative behavior, Gastroscopy, Risk factor

## Abstract

**Objective:**

To investigate the incidence of and risk factors associated with negative postoperative behavioral changes (NPOBCs) in children undergoing painless gastroscopy.

**Methods:**

Inclusion criteria: ASA I–II and outpatients aged 6–12 years undergoing painless gastroscopy. Exclusion criteria: history of surgery or anesthesia, children with developmental or intellectual abnormalities, refusal to participate, preoperative abdominal pain score > 3 points, history of chronic abdominal pain of > 3 months duration, and serious intraoperative complications. On the 1st, 14th, and 30th day after the gastroscopy, the Post Hospitalization Behavior Questionnaire for Ambulatory Surgery (PHBQ-AS) was used to assess NPOBCs in children.

**Results:**

A total of 1,670 children were included in this prospective observational cohort study. The incidence rates of NPOBCs were 14.13%, 4.55%, and 2.14% on the 1st, 14th, and 30th day after gastroscopy, respectively. The risk factors for the first day were female sex (OR 1.34, 95% CI 1.00–1.79), parental anxiety (OR 2.23, 95% CI 1.75–3.12), and severe anxiety in children (OR 2.83, 95% CI 1.96–4.07). The risk factors on the 14th day were parental anxiety (OR 3.71, 95% CI 2.19–6.29), a parental educational level above high school (OR 1.65, 95% CI 1.00–2.70), and severe anxiety in children (OR 11.87, 95% CI 5.85–24.07). The risk factors on the 30th day were female sex (OR 2.99, 95% CI 1.41–6.34), being an only child (OR 4.42, 95% CI 2.18–8.95), a parental educational level above high school (OR 2.66, 95% CI 1.27 NPOBCs 5.56), and severe anxiety in children (OR 6.84, 95% CI 2.84–16.49).

**Conclusion:**

In children undergoing painless gastroscopy, the incidence rates of NPOBCs on the 1st, 14th, and 30th day were 14.13%, 4.55%, and 2.14%, respectively. The risk factors for NPOBCs were severe anxiety in children, female sex, parental anxiety, and a parental educational level above high school. In particular, severe preoperative anxiety in children was a persistent risk factor for NPOBCs within 30 days.

## Introduction

Negative postoperative behavioral changes (NPOBCs) in children are well-recognized in the pediatric anesthesiologist community and have been studied for decades [1]. The incidence of NPOBCs varies greatly from 22.0 to 80.4% owing to the length of hospital stay, complexity of the surgery, and duration of anesthesia [1–8]. More importantly, postoperative pain is the most critical risk factor for NPOBCs [1, 4, 6, 9]. However, a large proportion of diagnostic procedures such as gastroscopy are painless. Although the duration of gastroscopy is generally within 30 min, it still requires sedation or anesthesia. In our tertiary public children’s hospital, more than 3,000 gastroscopic procedures are performed annually, and 61 similar public children’s hospitals exist in China. For this rather large population, it remains unknown whether short-term anesthesia for these painless procedures influences NPOBCs. We hypothesized that compared with complex surgical anesthesia, for painless procedures with short-term anesthesia, the incidence of NPOBCs might be low, but a large number of cases would be needed for an appropriate sample size. For this reason, we needed a simple, efficient, and reliable tool to assess NPOBCs. The Post Hospitalization Behavior Questionnaire for Ambulatory Surgery (PHBQ-AS) is an appropriate assessment tool for outpatient children, reducing the 27 items of the original PHBQ to 11 items [10]. We also hypothesized that in the absence of pain effects, the anxiety level of the child, the child’s sex, parental anxiety, and educational situation of the parents might be associated with NPOBCs. The objective of this study was to investigate the incidence and risk factors associated with NPOBCs in children undergoing painless gastroscopy.

## Methods

### Ethical approval

The Safety and Quality Management Committee at Kunming Children’s Hospital and the Kunming Ethics Committee approved this prospective observational cohort study (2020-03-070-K01), and the study was registered at the Chinese clinical trial registry (https://www.chictr.org.cn, registration number:ChiCTR2000034939). Written informed consent was obtained from the parents or legal guardians of all enrolled children.

### Study population

Inclusion criteria: outpatient children aged 6–12 years (ASA I–II) scheduled for gastroscopy at the Department of Sedation and Analgesia Center, Endoscopic Center of Kunming Children’s Hospital. Exclusion criteria: history of surgery or anesthesia, neurodevelopmental or mental disorders, parents or legal guardians of the patient refused consent for study participation, preoperative abdominal pain score > 3, chronic abdominal pain of > 3 months duration, and serious intraoperative complications, including severe (SPO_2_ < 75%, any duration) or prolonged (SPO_2_ < 90% for > 60 s) oxygen desaturation, apnea > 60 s, cardiovascular collapse/shock, cardiac arrest, or absent pulse [11].

### Preoperative assessment and anesthesia protocol

The preoperative anxiety levels of the children and their parents were separately assessed by an independent investigator in the waiting room. The modified Yale Preoperative Anxiety Scale (m-YPAS) was used to assess the preoperative anxiety level of the child. A score < 30 was defined as no anxiety, a score of 30–40 was defined as mild anxiety, and a score > 40 was defined as severe anxiety [12]. The visual analog scale (VAS) was used to assess parental anxiety; scores > 4 was defined as parental anxiety [13].

After baseline monitoring, administration of 2 L/min oxygen via a nasal catheter, and successful placement of a peripheral venous catheter, all children received 0.1 µg/kg sufentanil and intermittent boluses of propofol (2–3 mg/kg) intravenously. An experienced pediatric anesthesiologist was in charge of anesthesia and airway management. After completing the gastroscopy, the child was transferred to the postanesthesia care unit (PACU) until the Aldrete score was ≥ 9. The parents were encouraged to communicate with the child, and the family was allowed to leave the PACU after the assessment by a nurse anesthetist.

### Outcomes

The primary outcome was the incidence of NPOBCs. The PHBQ-AS has 11 items for 6 subscales, and each item has 5 degrees to score the postoperative behavioral changes of the children by their parents (Table 1) [10]. The presence of NPOBCs in this study was defined as at least one item being scored “more” or “much more” by the parents. The parents or guardians of the child were contacted by telephone for follow-ups by three investigators on the 1st, 14th, and 30th day after the gastroscopy.

**Table 1:** Post Hospitalization Behavior Questionnaire for Ambulatory Surgery (PHBQ-AS)

Subscale	Items
Eating	Does your child make a fuss about eating? Does your child spend time just sitting or lying and doing nothing? Does your child have a poor appetite?
General anxiety	Is your child uninterested in what goes on around him (or her)?
Separation anxiety	Does your child get upset when you leave him (or her) alone for a few minutes? Does your child have bad dreams at night or wake up and cry?
Apathy	Does your child need a lot of help doing things? Is it difficult to get your child interested in doing things (like playing games with toys)? Is it difficult to get your child to talk to you?
Aggression	Does your child have temper tantrums?
Sleep	Does your child have trouble getting to sleep at night?

All items were rated on a five-point scale labeled much less, less, the same, more, or much more

Secondary outcomes were variables predicting the occurrence of NPOBCs, including the preoperative anxiety levels of the children and parents, living environment, educational level of the parents, presence or absence of siblings, being a single parent, and age and sex of the child.

### Statistical analysis

All statistical analyses were performed using SPSS software version 23.0 (SPSS Inc.). Normally distributed continuous variables are expressed as mean ± standard deviation (SD); categorical variables are expressed as frequency and percentage. Univariate analysis was conducted for each variable using the chi-square test. Moreover, multivariate analysis was performed using multiple logistic regression to identify potential independent risk factors for NPOBCs. The results are presented as odds ratios (ORs) and 95% confidence intervals (CIs). *P* < 0.05 was considered to indicate statistically significant differences.

An online tool (https://www.powerandsamplesize.com/) was used to calculate the sample size, according to the early follow-up results. Among 339 children who completed the follow-up within 60 days, the incidence of NPOBCs was 4.72%. We, thus, hypothesized that the expected incidence of NPOBCs was 3%. According to this incidence, 1,032 children would be needed to meet the following conditions: (1-β) = 90%, α = 0.05, 95% CI 0.016–0.048. We assumed a dropout rate of 10%; therefore, we planned to follow up for 1 year to increase the sample size to over 2,000 children.

## Results

### Patient selection process

**Fig. 1:**
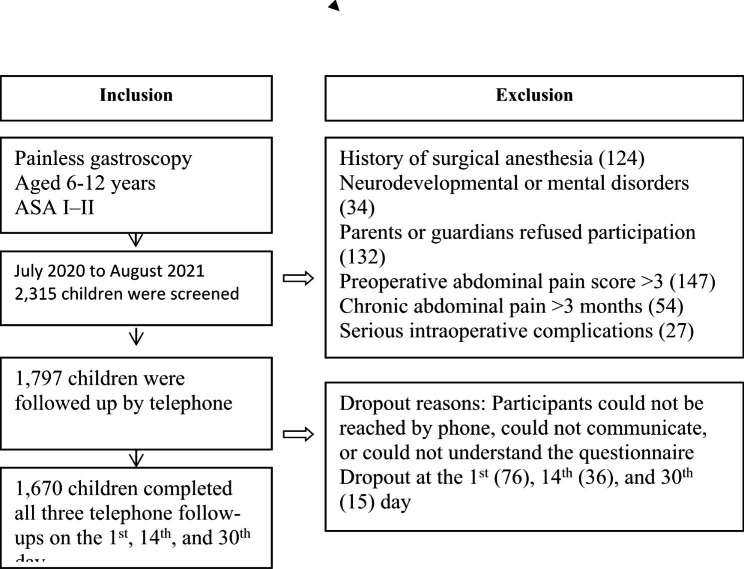
Study enrollment

From July 2020 to August 2021, 2,315 children and their families were screened. Of these, 518 children were excluded, and the remaining 1,797 children were followed up by telephone. In total, 127 children were lost to follow-up, with a dropout rate of 7.63%. Thus, 1,670 participants completed all three telephone follow-ups on the 1st, 14th, and 30th day after gastroscopy and were included in the study (Fig. 1).

### Patient characteristics

The age was 9.25 ± 2.14 years old(Table 2). Parental educational level above high school was 35.45%; parental anxiety was 36.11%. Mild anxiety in children was 43.59%, severe anxiety in children was 16.11%. Induction time was 5.39 ± 0.93 min, procedure time was 8.07 ± 1.59 min, recovery time was 14.91 ± 4.20 min. The gastritis was 72.16%, and negative diagnosis was 10.24 in the post-gastroscopy diagnosis.

**Table 2:** Patient characteristics

Characteristics	N = 1670	(%)		
**Age (year)**	9.25 ± 2.14			
**Gender**				
Male	888	53.17		
Female	782	46.93		
**Parents**				
Single parent	69	4.13		
Two parents	1601	95.87		
**Siblings**				
Only child	354	21.20		
Sibling child	1316	78.80		
**Parental anxiety(VAS)**				
Yes (VAS>4)	603	36.11		
No (VAS ≤ 4)	1067	64.01		
**Living environment**				
Urban	649	38.86		
Rural	1021	61.14		
**Parental educational level**				
<High school	1078	64.55		
>High school	592	35.45		
**Anxiety level**(m-YPAS)				
No(<30)	673	40.30		
Mild(30–40)	728	43.59		
Severe(>40)	269	16.11		
**Anesthesia**				
Propofol (mg)	87.75 ± 27.18			
Sufentanil (µg)	2.76 ± 0.85			
Induction time (min)	5.39 ± 0.93			
Procedure time (min)	8.07 ± 1.59			
Recovery time (min)	14.91 ± 4.20			
**Post-gastroscopy diagnosis**				
Gastritis	1205		72.16	
Helicobacter pylori infection	174		10.42	
Gastroesophageal reflux	53		3.17	
Gastric/duodenal ulcer	18		1.08	
Others	49		2.93	
Negative diagnosis	171		10.24	

Induction time: the time from intravenous propofol and sufentanil to the start of gastroscopy; procedure time: the time from the start of gastroscopy to completion of gastroscopy; recovery time: the time from completion of gastroscopy to attainment of the standard of recovery

### Incidence of NPOBCs on the first day and risk factor analysis

The incidence of NPOBCs on the first day was 14.13% (236/1,670, 95% CI 12.46–15.80; Table 3). The univariate analysis found that female sex, being an only child, parental anxiety, and severe anxiety in children were significantly associated with NPOBCs. The multivariate logistic regression found that sex (OR 1.34, 95% CI 1.00–1.79), parental anxiety (OR 2.23, 95% CI 1.75–3.12), and severe anxiety in children (OR 2.83, 95% CI 1.96–4.07, versus no anxiety) were independent predictors of NPOBCs.

**Table 3:** Incidence of NPOBCs on the first day and risk factor analysis

Categories			Univariate		Multivariate	
N = 1670	NPOBCs N = 236 (14.13, 12.46–15.80)	No NPOBCs N = 1434	OR (95% CI)	*P*-value	OR (95% CI)	*P*-value
Sex						
Male (N = 888)	105 (44.5)	783 (54.6)	0.706 (0.556–0.896)	0.004^*a^	1.34 (1.00–1.79)	0.044^*b^
Female (N = 782)	131 (55.5)	651 (45.4)				
Parents						
Single parent (N = 69)	0 (0.0)	69 (4.8)	**-** -			
Two parents (N = 1601)	236(100.0)	1365 (95.2)				
Siblings						
Only child (N = 354)	35 (14.8)	319 (22.2)	0.647(0.461–0.909)	0.010^*a^		
Sibling child (N = 1316)	201 (85.2)	1115 (77.8)				
Parental anxiety						
Yes (N = 603)	129 (54.7)	474 (33.1)	2.133(1.685–2.701)	<0.001^*a^	2.23 (1.75–3.12)	<0.001^*b^
No (N = 1067)	107 (45.3)	960 (66.9)				
Living environment						
Urban (N = 649)	80 (33.9)	569 (39.7)	0.087 (0.628–1.037)	0.091^a^		
Rural (N = 1021)	156 (66.1)	865 (60.3)				
Parental educational level						
<High school (N = 1078)	157 (66.5)	921 (64.2)	1.091 (0.849–1.403)	0.494^a^		
>High school (N = 592)	79 (33.5)	513 (35.8)				
Anxiety level(m-YPAS)						
No<30 (N = 673)	80 (33.9)	593 (41.4)				
Mild 30–40(N = 728)	77 (32.6)	651 (45.4)				
Severe >40 (N = 269)	79 (35.5)	190 (13.2)		<0.001^*a^	2.83 (1.96–4.07)	<0.001^*b^

CI, confidence interval; NPOBCs, negative postoperative behavioral changes; OR, odds ratio; m-YPAS, modified Yale preoperative anxiety scale, a score < 30 was defined as no anxiety, a score of 30–40 was defined as mild anxiety, and a score > 40 was defined as severe anxiety. a Pearson chi-square test. b multiple logistic regression

### Incidence of NPOBCs on the 14th day and risk factor analysis

The incidence of NPOBCs on the 14th day was 4.55% (76/1,670, 95% CI 3.55–5.55%; Table 4). The univariate analysis found that being an only child, parental anxiety, a parental educational level above high school, and severe anxiety in children were associated with NPOBCs. Multivariate logistic regression found that parental anxiety (OR 3.71, 95% CI 2.19–6.29), a parental educational level above high school (OR 1.65, 95% CI 1.00–2.70), and severe anxiety in children (OR 11.87, 95% CI 5.85–24.07, versus no anxiety) were independent predictors of NPOBCs.

**Table 4:** Incidence of NPOBCs on the 14th day and risk factor analysis

Categories			Univariate		Multivariate	
N = 1670	NPOBCs N = 76 (4.55, 3.55–5.55)	No NPOBCs N = 1594	OR (95% CI)	*P*-value	OR (95% CI)	*P*-value
Sex						
Male(N = 888)	35 (46.1)	853 (53.5)	0.752(0.484–1.168)	0.203 ^a^		
Female (N = 782)	41 (53.9)	741 (46.5)				
Parents						
Single parent (N = 69)	5 (6.6)	64 (4.0)	1.634(0.682–3.917)	0.422 ^a^		
Two parents (N = 1601)	71 (93.4)	1530 (96.0)				
Siblings						
Only child (N = 354)	25 (32.9)	329 (20.6)	1.822(1.146–2.898)	0.011^*a^		
Sibling child (N = 1316)	51(67.1)	1265(79.4)				
Parental anxiety						
Yes (N = 603)	54(71.1)	549(34.4)	4.343(2.673–7.058)	<0.001^*a^	3.71(2.19–6.29)	<0.001^*b^
No (N = 1067)	22(28.9)	1045(65.6)				
Living environment						
Urban (N = 649)	35(46.1)	614(38.5)	1.343(0.865–2.086)	0.188 ^a^		
Rural (N = 1021)	41 (53.9)	980 (61.5)				
Parental educational level						
<High school (N = 1078)	37 (48.7)	1041 (65.3)	0.521 (0.336–0.808)			
>High school (N = 592)	39(51.3)	553 (34.7)		0.003^*a^	1.65 (1.00–2.70)	0.048^*b^
Anxiety level(m-YPAS)						
No<30 (N = 673)	10 (13.2)	663 (41.6)				
Mild 30–40 (N = 728)	17(22.4)	711 (44.6)				
Severe >40(N = 269)	49 (64.5)	220(13.8)		<0.001^*a^	11.87 (5.85–24.07)	<0.001^*b^

CI, confidence interval; NPOBCs, negative postoperative behavioral changes; OR, odds ratio; m-YPAS, modified Yale preoperative anxiety scale, a score < 30 was defined as no anxiety, a score of 30–40 was defined as mild anxiety, and a score > 40 was defined as severe anxiety. a Pearson chi-square test. b multiple logistic regression

### Incidence of NPOBCs on the 30th day and risk factor analysis

The incidence of NPOBCs on the 30th day was 2.14% (39/1,670; 95% CI 1.61–3.06%; Table 5). The univariate analysis found that female sex, being an only child, a parental educational level above high school, and severe anxiety in children were significantly associated with NPOBCs. The multivariate logistic regression found that female sex (OR 2.99, 95% CI 1.41–6.34), being an only child (OR 4.42, 95% CI 2.18–8.95), a parental educational level above high school (OR 2.66, 95% CI 1.27–5.56), and severe anxiety in children (OR 6.84, 95% CI 2.84–16.49, versus no anxiety) were independent predictors of NPOBCs.

**Table 5:** Incidence of NPOBCs on the 30th day and risk factor analysis

Categories			Univariate		Multivariate	
N = 1670	NPOBCs N = 39 (2.34,1.61–3.06)	No NPOBCs N = 1631	OR (95% CI)	*P*-value	OR (95% CI)	*P*-value
Sex						
Male (N = 888)	10 (25.6)	878 (53.8)	0.294(0.144–0.597)			
Female (N = 782)	29 (74.4)	753 (46.2)		<0.001^*a^	2.99 (1.41–6.34)	0.004^*b^
Parents						
Single parent (N = 69)	0 (0.0)	69 (4.2)				
Two parents (N = 1601)	39 (100.0)	1562 (95.8)				
Siblings						
Only child (N = 354)	24 (61.5)	330 (20.2)	5.95 (3.315–11.22)	<0.001^*a^	4.42 (2.18–8.95)	<0.001^*b^
Sibling child (N = 1316)	15 (38.5)	1265 (79.8)				
Parental anxiety						
Yes (N = 603)	14 (35.9)	589 (36.1)	0.953(0.501–1.811)	0.978 ^a^		
No (N = 1067)	25 (64.1)	1042 (63.9)				
Living environment						
Urban (N = 649)	13 (33.3)	636 (39.0)	0.847(0.446–1.610)	0.612 ^a^		
Rural (N = 1021)	26 (66.7)	995 (61.0)				
Parental educational level						
<High school (N = 1078)	12 (30.8)	1066 (65.4)	0.244(0.125–0.478)			
>High school (N = 592)	27 (69.2)	565 (34.6)		<0.001^*a^	2.66 (1.27–5.56)	0.009^*b^
Anxiety level(m-YPAS)						
No <30 (N = 673)	7 (17.9)	666 (40.8)	**-**	<0.001^*a^		
Mild 30–40 (N = 728)	9 (23.1)	719 (44.1)				
Severe >40(N = 269)	23 (59.0)	246 (15.1)			6.84 (2.84–16.49)	<0.001^*b^

CI, confidence interval; NPOBCs, negative postoperative behavioral changes; OR, odds ratio; m-YPAS, modified Yale preoperative anxiety scale, a score < 30 was defined as no anxiety, a score of 30–40 was defined as mild anxiety, and a score > 40 was defined as severe anxiety. a Pearson chi-square test. b multiple logistic regression

### NPOBC subscales

**Fig. 2:**
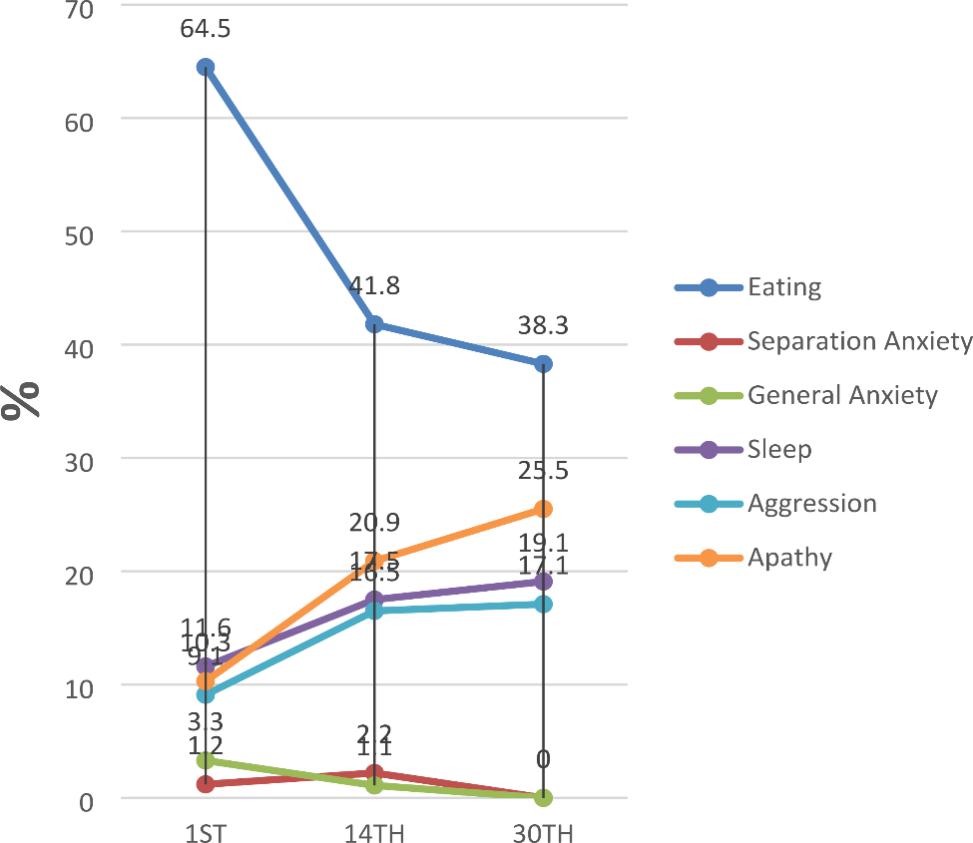
Proportions of NPOBC subscales on the 1st, 14th, and 30th day after the gastroscopy NPOBCs, negative postoperative behavioral changes

On the 1st, 14th, and 30th day, eating problems were reported in 156 (64.46%), 38 (41.76%), and 18 (38.30%) children, respectively. Problems with sleeping were found in 28 (11.57%), 16 (17.58%), and 9 (19.14%) children, aggression in 22 (9.09%), 15 (16.48%), and 8 (17.02%) participants, apathy in 25 (10.33%), 19 (20.88%), and 12 (25.53%) children, separation anxiety in 3 (1.24%), 2 (2.20%), and 0 children, and general anxiety in 8 (3.31%), 1 (1.10%), and 0 participants, respectively (Fig. 2).

.

## Discussion

Between July 2020 and August 2021, 1,670 participants completed three telephone follow-ups. According to our data from children undergoing painless gastroscopy, the incidence rates of NPOBCs on the 1st, 14th, and 30th day were 14.13%, 4.55%, and 2.14%, respectively. The risk factors for NPOBCs were severe anxiety in children, female sex, parental anxiety, and a parental educational level above high school. In particular, severe preoperative anxiety in the child was a persistent risk factor for NPOBCs within 30 days. In the NPOBC subscales, eating problems accounted for the largest proportion but showed a decreasing trend, followed by sleep problems, aggression, and apathy all of which showed an increasing trend over time. Separation anxiety and general anxiety accounted were the least important.

The incidence rates of NPOBCs in this study (2.14–14.13%) were considerably lower than those of previous studies (22.0–80.4%) [2–4]. A major difference from previous studies is that our study excluded the effects of pain, and previous studies have shown that pain is an essential risk factor for NPOBCs [1, 4, 6, 9]. For this particular reason, we chose gastroscopy as an appropriate inclusion criterion while excluding children with a preoperative abdominal pain score > 3. As expected, the incidence of NPOBCs in this “pain-free” group of children is extremely low. Moreover, our study excluded the effects of prolonged hospitalization. Stargatt et al. found that staying two nights or more in a hospital is a risk factor associated with NPOBCs [3], and children undergoing ambulatory surgery have fewer psychological disorders [14]. Thus, previously healthy outpatient children undergoing gastroscopy may have a lower incidence of NPOBCs than children with long-term hospitalization or chronic diseases. An additional reason for the lower incidence rate in our study might be the age of the study population. Younger age, especially under 4 years, is a risk factor for NPOBCs [1–3], [7]. The average age of children in our study was 9 years, and negative behaviors in older children may have been ignored by family members or masked.

Among the identified risk factors for NPOBCs, the external factors of parental educational level and preoperative anxiety affect the occurrence of negative behaviors; this is similar to findings of previous studies [3, 9], [15–18]. However, these risk factors only appear at a certain time point and have no continuous impact on the children’s behaviors. Unlike previous studies [15, 16, 19], our study found that severe, but not mild, anxiety is a risk factor for NPOBCs. Interestingly, the persistent existence of severe anxiety has a continuous impact on children’s behaviors. An assessment of temperament may help identify pediatric patients at risk of preoperative anxiety [20]. Thus, temperament may not only cause preoperative anxiety but also lead to long-term NPOBCs.

To our knowledge, this is the first study to describe the proportions of NPOBC subscales. We found that eating problems accounted for the largest proportion, this may be affected by the digestive tract disorder, but the proportion of eating problems showed a decreasing trend, a possible reason is that the digestive tract disorder improves after a period of treatment. Separation anxiety and general anxiety accounted for the lowest proportions, possibly because the three questions associated with these subscales rarely have negative answers for older children. For example, the question “Does your child get upset when you leave him/her alone for a few minutes?” is usually answered with “No” for older children. We also found that the proportions of apathy, aggression, and sleep problems gradually increased. These negative behaviors may lead to more serious psychological and behavioral problems.

This study has some limitations. First, because there were no non-anesthesia gastroscopy patients in our hospital, we did not compare the difference of NPOBCS between non-anesthesia and anesthesia patients. Second, although Jenkins et al. confirmed both the validity and consistency of the PHBQ-AS [10], the PHBQ-AS itself has certain limitations and controversies [21, 22]. Owing to the large sample size of our study, the PHBQ-AS with its simplicity and efficiency was the best choice for this study. Third, a further study should focus on whether the preoperative characteristics or temperament of the child is associated with the categorization of negative behavioral changes.

## Conclusion

The incidence rates of NPOBCs in children undergoing painless gastroscopy on the 1st, 14th, and 30th day were 14.13%, 4.55%, and 2.14%, respectively. The risk factors for NPOBCs were severe anxiety in children, female sex, parental anxiety, and a parental educational level above high school. In particular, severe preoperative anxiety in children was a persistent risk factor for NPOBCs within 30 days. Among NPOBC subscales, eating problems accounted for the largest proportion but showed a decreasing trend, followed by sleep problems, aggression, and apathy which all showed increasing trends, whereas separation anxiety and general anxiety accounted for the lowest proportions.

## Data Availability

The data that support the findings of this study are available from corresponding author [HuangLei] but restrictions apply to the availability of these data, which were used under license for the current study, and so are not publicly available. Data are however available from the authors upon reasonable request and with permission of [HuangLei].

## Abbreviations

NPOBCS: Negative postoperative behavioral changes
ASA: American society of anesthesiologists
PHBQ-AS: Post hospitalization behavior questionnaire for ambulatory surgery
SPO_2_: Pulse oximeter oxygen saturationm-YPAS Modified yale preoperative anxiety scale
VAS: Visual analog scale
PACU: Postanesthesia care unit
